# Evaluation of cell metabolic adaptation in wound and tumour by Fluorescence Lifetime Imaging Microscopy

**DOI:** 10.1038/s41598-020-63203-4

**Published:** 2020-04-14

**Authors:** Diego Morone, Francesca D’ Autilia, Tilo Schorn, Marco Erreni, Andrea Doni

**Affiliations:** 1 Unit of Advanced Optical Microscopy, IRCCS, Humanitas Clinical and Research Center, Rozzano, Milan Italy; 2 Present Address: Faculty of Biomedical Sciences, Institute for Research in Biomedicine, Università della Svizzera italiana (USI), Bellinzona, Switzerland

**Keywords:** Biological techniques, Cell biology

## Abstract

Acidic pH occurs in acute wounds progressing to healing as consequence of a cell metabolic adaptation in response to injury-induced tissue hypoperfusion. In tumours, high metabolic rate leads to acidosis affecting cancer progression. Acidic pH affects activities of remodelling cells *in vitro*. The pH measurement predicts healing in pathological wounds and success of surgical treatment of burns and chronic ulcers. However, current methods are limited to skin surface or based on detection of fluorescence intensity of specific sensitive probes that suffer of microenvironment factors. Herein, we ascertained relevance *in vivo* of cell metabolic adaptation in skin repair by interfering with anaerobic glycolysis. Moreover, a custom-designed skin imaging chamber, 2-Photon microscopy (2PM), fluorescence lifetime imaging (FLIM) and data mapping analyses were used to correlate maps of glycolytic activity *in vivo* as measurement of NADH intrinsic lifetime with areas of hypoxia and acidification in models of skin injury and cancer. The method was challenged by measuring the NADH profile by interfering with anaerobic glycolysis and oxidative phosphorylation in the mitochondrial respiratory chain. Therefore, intravital NADH FLIM represents a tool for investigating cell metabolic adaptation occurring in wounds, as well as the relationship between cell metabolism and cancer.

## Introduction

In normal skin, dermis has an extracellular neutral-alkaline pH^1,2^. After tissue damage, cells involved in tissue repair undergo a metabolic adaptation resulting from low oxygen tension towards a less energy-efficient process of anaerobic metabolism that leads to microenvironment acidosis^2,3^. *In* *vitro* evidence suggests a beneficial effect of acidic pH in several processes implicated in wound healing, which involve cell adhesion^4,5^, migration and proliferation^6^. In a previous report^7^, we described an unprecedented role of tissue acidification in setting PTX3, a key component of the humoral arm of innate immunity, in a tissue remodelling and repair mode. In wounds, reduced oxygen tension^8^ and factors downstream of cell anaerobic glycolysis, such as lactate^9,10^, effectively stimulate immune and vascular endothelial cells to release factors that support angiogenesis. Subsequent neovascularization allows restoration of nutrient delivery and oxygen, and cells use oxidative metabolism for their longer-term functions contributing to restore the wound pH to values near to neutral^2,3^.

While an acidic pH occurs in the inflammatory phases of acute wounds that progress on healing, chronic and highly infected wounds are characterized by abundant recruitment of neutrophils and a non-acidic pH^11,12^. Chronic non-healing wounds may occur secondarily to a high alkaline pH^11,13^, and studies report a relationship between wound pH and chronic wound healing^14–16^. The effect of acidic pH in the wound bed has a clinical functional relevance on the healing of chronic wounds^11^. In fact, a prolonged chemical acidification of the wound bed increases the healing rate in chronic venous leg ulcers^17^. Chronic non-healing wounds continue to represent a therapeutic challenge for clinicians. Indeed, measurement of wound pH predicts healing outcomes and skin graft survival in experimental and clinical studies and determines success or failure of surgical treatment of burns and chronic ulcers^11,17^. Measurement of skin pH is usually carried out with pH electrodes, which is limited to the skin surface or in the evaluation of superficial environmental factors^13^ and provides an inherently low spatial resolution. The measurement in the inner parts of the skin can be attempted by the subsequent removal of skin layers with adhesive tape^18^, however applied with limitation to measurement in the damaged dermis.

In tumours, a high metabolic rate leads to acidosis in poorly perfused regions as a result of high glucose consumption and high lactate production^9,19,20^, thus affecting cancer progression^10,21–23^. Acidic tumour microenvironment induces cancer cells to increase formation of lamellipodia, adhesion and invasiveness, as well as the increased secretion of ECM proteases^24–27^. A possible molecular mechanism underlying the promotion of cell adhesion and invasion was related to the pH-dependent activation of cell surface integrins^5^. Interference with pH may represent a therapeutic target also in cancer^28^. Macrophages have a direct impact on metabolism in the tumour microenvironment^10,29^. M1-polarized macrophages show a metabolic shift towards an anaerobic glycolytic pathway typical in acute infections and hypoxic tissues, and iron storage through ferritin expression. On the other hand, M2-polarized macrophages show an oxidative glucose metabolism and express high levels of ferroportin, an iron exporter that promotes tissue repair and tumour growth^29,30^. The definition of mechanisms that regulate the metabolic activity of macrophages may be important to define their relevance in cancer progression^10^.

Herein, we investigated the actual relevance *in vivo* of cellular metabolic adaptation in a skin repair model and we applied *in vivo* 2PM, FLIM and mapping analysis for the measurement of NADH spatial distribution; we then correlated these results with parameters of acidification and hypoxia of the microenvironment, thus providing a method to assess the cellular metabolic adaptation occurring in tissue repair, as well as in cancer.

## Results

### Relevance of cell metabolic adaptation in wound healing *in vivo*

**Figure 1:**
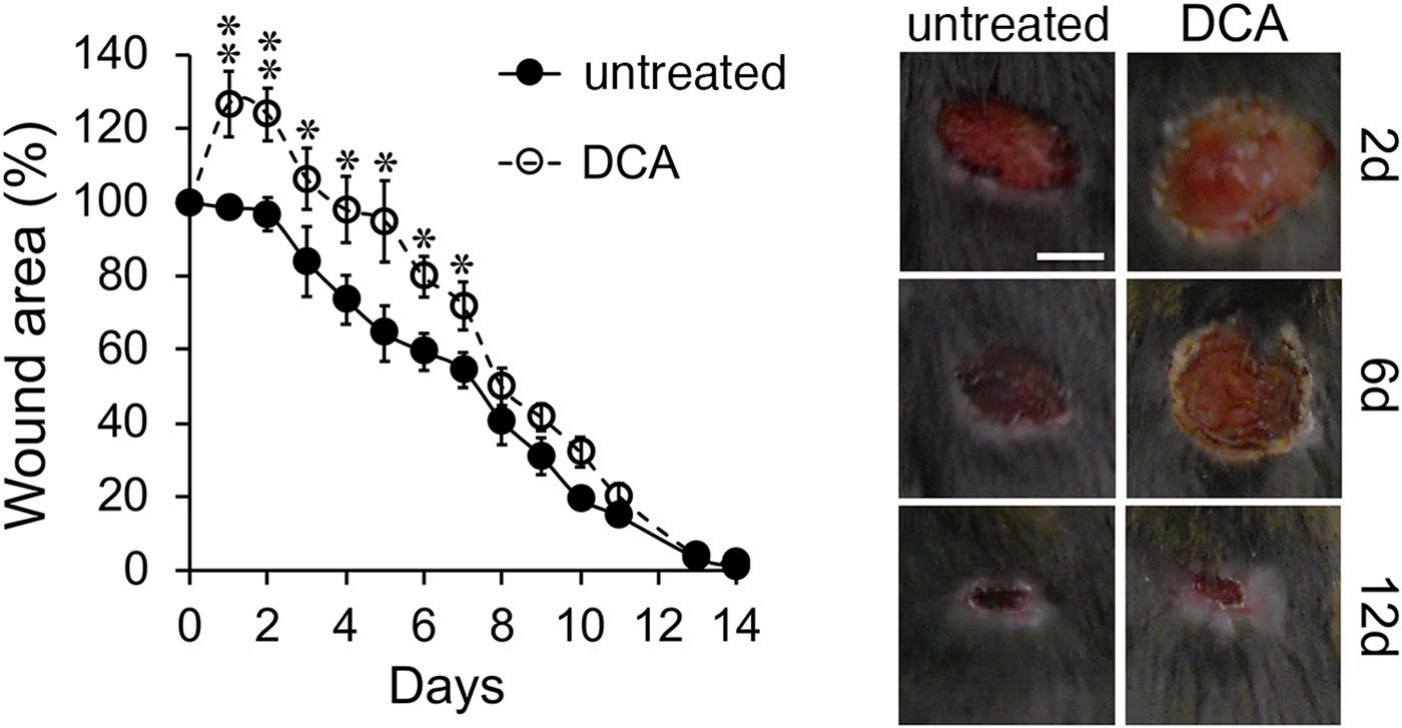
*In vivo* relevance of the cell metabolic adaptation in skin repair. The excisional skin wound healing model was used. Left, kinetic analysis of skin wound areas was performed. Values represent mean ± SD. Right, representative macroscopic images of untreated and DCA treated mice are shown at the indicated days after wounding. Bar, 5 mm. *P < 0.05, **P < 0.01; Student’s *t*-test. Untreated, n = 8; DCA, n = 9. One experiment shown out of two performed with similar number of animals.

Evidences suggest that wound acidification resulting from the cell metabolic adaptation after damage acts as a regulator of several activities involved in tissue repair^11,14,16^. In order to determine the relevance of wound acidification in healing outcome *in vivo*, the pyruvate dehydrogenase kinase (Pdk) inhibitor dichloroacetate (DCA) was used in a model of full-thickness excisional skin wounding to promote glucose oxidation over anaerobic glycolysis, thus interfering with local acidosis. As shown in Fig. 1, mice treated (n = 9) with a single administration of DCA (300 mg/Kg) 1 h prior skin wounding showed significantly delayed curve compared to mice treated with vehicle alone (n = 8). As assessed by measurement of wound area, DCA-mediated delayed wound closure corresponded to: day 1, −28.3% (P = 0.005); day 2, −27.1% (P = 0.004); day 3, −22.5% (P = 0.05); day 4, −24.5% (P = 0.02); day 5, −30.5% (P = 0.002); day 6, −20.4% (P = 0.01); day 7, −17.4% (P = 0.03); compared to control mice (Fig. 1), thus inhibition of cellular anaerobic glycolysis at early stages significantly affects the process of tissue repair.

### Measurement of cell glycolysis activity in wound by FLIM

Relevance of the results *in vivo* prompted us to draft a method for investigating the cellular metabolic adaptation that occurs in tissue repair. Anaerobic glycolysis leads to an imbalance of the metabolic coenzyme NADH, as main electron exchanger in the glucose cycle, over NAD^+^. However, NADH loses fluorescence intensity upon oxidation to NAD^+^. Furthermore, fluorescence intensity measurements are subject to variations in concentration and quantum yield differences between free and protein-bound forms of NADH. Therefore, the fluorescence lifetime decay of the metabolic coenzyme NADH was measured to evaluate the cell glycolysis activity in wounded skin *in vivo*. An experimental model of dorsal full-thickness incisional wounding^7^ and a custom-designed imaging chamber (Fig. S1) were used for the purpose. This chamber allowed us to visualize the dorsal skin mounted flat inside an imaging window from the epidermis to deeper tissue (Fig. S2) without any surgical intervention: of note, previously reported skinfold chambers aimed at visualizing skin vasculature^31^ and subcutaneous tumours^32^ require surgery, which would alter the measurement associated with the cellular metabolic changes induced by tissue damage. The skinfold chamber can be easily disassembled after imaging to reduce animal stress and to perform subsequent acquisitions over time.

**Figure 2:**
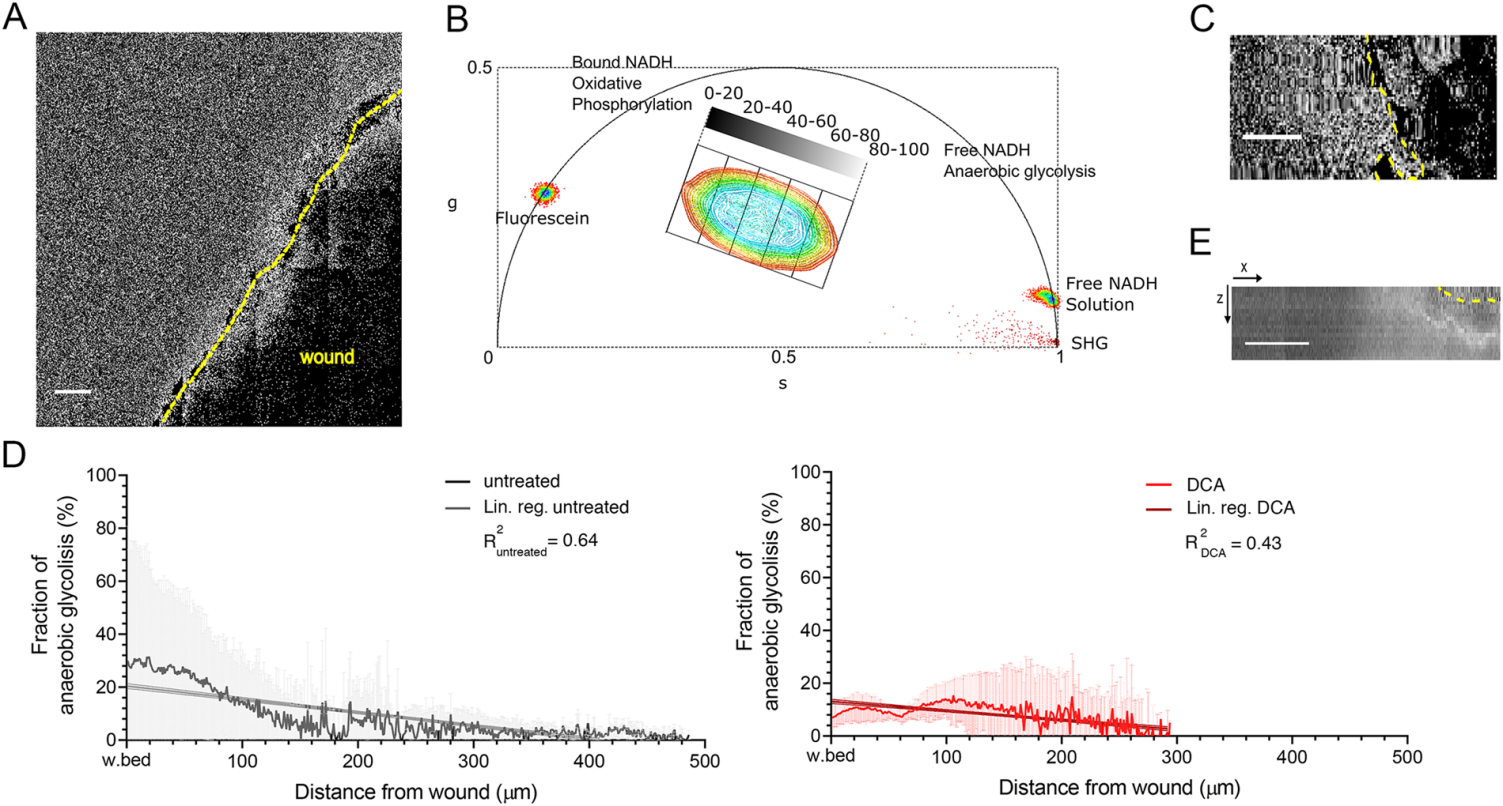
Measurement of anaerobic glycolysis in wound by FLIM quantification of NADH/NAD^+^ ratio. Phasor analysis of free and bound NADH fluorescence allows a quantification of their relative increase. (**A**) Map of a gradient of NADH/NAD^+^ ratio (grey). Wound margin is highlighted with a yellow dashed line. All scale bars 100 µm. (**B**) Phasor analysis of FLIM NADH fluorescence. NADH region of the phasor plot was divided in five sub-regions along the major axis and each is associated with an intensity value (0–20, 20–40, 40–60, 60–80, 80–100). Reference calibration points for SHG, to account for the IRF, and for fluorescein and NADH are also displayed (n = 5 measurements for each performed). (**C**) The image shows a representative portion of a straightened image of NADH/NAD^+^ ratio from wound margin (yellow dashed line). (**D**) NADH/NAD^+^ ratio percentage as a function of distance from wound (grey). The graph shows the increase of NADH production around the wound. Linear regression with 95% confidence level bands (black line) shows a significant decrease in the ratio from the wound front (Deviation from slope zero test, P < 0.0001); n = 11 mice. NADH/NAD^+^ ratio is lower in perilesional area of DCA-treated mice (red line vs grey line, comparison between first 10 values of each condition, *t*-test ****P < 0.0001). n = 3 mice. Comparison between linear regressions with 95% confidence level bands (darker lines with bands) show a reduction in line slope in DCA-treated mice (Deviation between slopes test, P < 0.0001. Deviation from slope zero test, P < 0.0001). (**E**) *XZ* maximum intensity projection shows a preferential increase of anaerobic glycolysis (higher intensity) around the wound margin.

On day 1 after wounding, FLIM images were acquired with 2PM and stitched to produce lifetime *XYZ* mosaics (600–1000 µm wide and 200 µm deep) (Fig. 2A). Common FLIM imaging has slow acquisition times, which are required to collect enough photons to reconstruct a time decay curve. This was compensated by the use of a time correlated single-photon counting (TCSPC) 16-channel parallel detector, with an average counting speed of 80 MHz which allowed faster 3D scanning of the mosaic over lesion area^33^. The ratio of free to protein-bound NADH has been known to be associated with the ratio of NAD^+^ to NADH in small intestine epithelial cells^34^ and breast cancer cells^35^. Protein-bound NADH has a complex multi-exponential lifetime decay that has been related to its binding to different enzymes, such as malate dehydrogenase and lactate dehydrogenase^36^. Standard approaches revolved on a characterization by fitting with a two-exponential curve, corresponding to a short or long lifetime range. This approximation is however misleading because free and protein-bound NADH have common exponential components and the NADH binding sites with different enzymes cannot be considered^34,37^. In fact, in our case this approach was ineffective to discriminate between untreated and DCA-treated mice (Fig. S3). Lifetime decays in perilesional regions of untreated and DCA-treated mice could not show significant differences when fitted with a two-exponential decay function (Fig. S3; Student *t*-test, P > 0.05). Instead, phasor analysis^34,38^ was used. In accordance with a previous report^39^, we designed an analysis that first divides the phasor cloud into five regions (Fig. 2B) corresponding to a gradient of prevailing anaerobic glycolysis in regions of lower lifetimes towards a region where more oxidative phosphorylation occurs. Pixels attributed to each region were then associated to discrete values of decreasing intensity, and therefore quantification of pixel intensity on the resulting image was proportional to the relative increase in anaerobic glycolysis. By linearizing images, we obtained a spatial distribution of anaerobic glycolysis from wound profile (Fig. 2C). At lesion margin, gradient of NADH/NAD^+^ ratio corresponded to 29.1 ± 0.4% and decreased until to 2.6 ± 0.1% distance (range of 200–500 μm) from the wound. The decrease was evaluated with a linear regression fitting as the simplest fitting model for testing the overall deviation from zero slope curve (linear regression fitting with a slope of −0.050 ± 0.0016 μm^−1^; black line with 95% CL bands; deviation from zero slope test, P < 0.0001) (Fig. 2d). An increased anaerobic glycolysis around wound margins was also evident in *XZ* maximum intensity projection images of the maps (Fig. 2e). Administration of DCA (300 mg/Kg, i.p.; 1 h) altered the glycolysis activity in wounds as assessed by phasor analysis of NADH (Fig. 2D, red). FLIM images were acquired in wounds of untreated mice and after injection of DCA. Reduction in anaerobic glycolysis at perilesional area resulted from 29.1 ± 0.4% to 8.0 ± 0.2% (mean of the first 10 values, *t*-test P < 0.0001). Also, gradient steepness evaluated with linear regression fitting was lowered from a slope of −0.050 ± 0.0016 μm^−1^ to −0.035 ± 0.0023 μm^−1^ (Fig. 2D, darker lines with 95% CL bands. Deviation between slopes test, P < 0.0001. Deviation from zero slope test for both conditions, P < 0.0001). To assess the effect of the mitochondrial respiratory chain on glycolytic gradient in wound, Rotenone and Antimycin A were used *in vivo* as inhibitors of the complexes I and III of the Electron Transport Chain (ETC), respectively. Rotenone was reported to reduce NADH lifetimes and increase accumulation of free NADH^40^. Antimycin A binds to the Qi site of cytochrome c reductase affecting the terminal respiratory chain and hence ATP production. Local administration of Rotenone (20 µM) impaired the formation of a gradient of NADH/NAD^+^ ratio, lowering the slope from −0.267 ± 0.06 μm^−1^ to −0.113 ± 0.002 μm^−1^ (Fig. S4, darker lines with 95% CL bands. Deviation between slopes test, P < 0.0001, n = 3 per group). Rotenone did not affect the fraction of anaerobic glycolysis at wound margin (from 20.28 ± 0.41% to 20.08 ± 0.21%, Student *t*-test P > 0.05) but it increased glycolysis in regions distant from the wound where an oxidative phosphorylation possibly occurs. Evidence are in line with previous reports^41,42^, where Rotenone leads a metabolic shift towards NADH accumulation and glycolysis from oxidative phosphorylation. Antimycin A (5 µM) altered the formation of a gradient (Fig. S4, slope decreased to −0.046 ± 0.08 μm^−1^; deviation between slopes test, P < 0.0001, untreated, n = 3; Antimycin A, n = 2) affecting, as expected^43^, glycolysis at wound margin (8.37 ± 0.49% (Student *t*-test, P < 0.0001).

In the same wound model, the classical probes Hypoxyprobe Pimonidazole-HCl (Pimo) and 2′,7′-Bis-(2-Carboxyethyl)-5-(and-6)-Carboxyfluorescein (BCECF) were used to correlate glycolytic activity with regions of hypoxia and extracellular acidic pH, respectively, in wounds.

**Figure 3:**
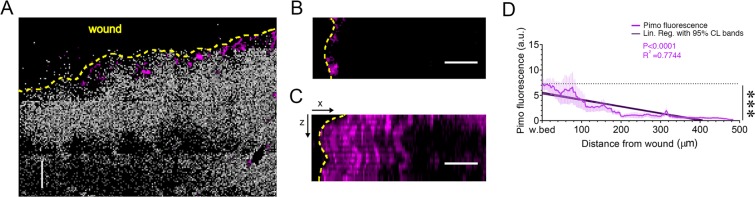
Analysis of hypoxia in wound. (**A**) The image shows a preferential localization of hypoxia (magenta) in regions of higher glycolytic metabolism (increased NADH/NAD^+^ ratio; grey). Pimonidazole (Pimo) fluorescence intensity was used to detect hypoxic regions. Wound margin is highlighted with a yellow dashed line. All scale bars 100 µm. (**B**) Pimo image straightened along wound margin (yellow dashed line). (**C**) *XZ* maximum intensity projection of Pimo fluorescence intensity. (**D**) Pimo intensity as a function of distance from the perilesional area. Linear regression 95% confidence level bands show a significant decrease (Deviation from slope zero test, P < 0.0001). n = 3 mice.

As assessed by measurement of Pimo fluorescence intensity, hypoxic areas were preferentially associated with increased NADH/NAD^+^ ratio (Fig. 3A) at wound margins (Fig. 3B,C). Linear regression of Pimo fluorescence intensity fitted with a slope of −0.0134 ± 0.00032 μm^−1^ (Fig. 3D, darker purple line with 95% CL bands. Deviation from zero slope test, P < 0.0001).

**Figure 4:**
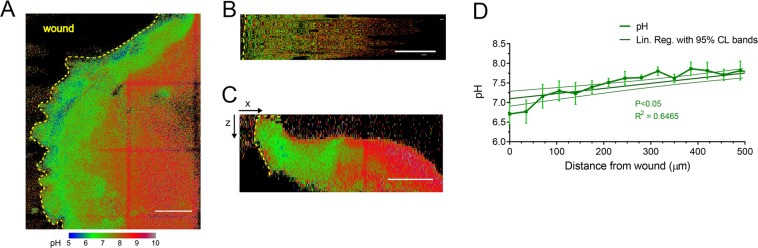
Analysis of pH in wound. (**A**) Maximum intensity projection of a representative pH map stack. False colours represent pH as indicated in the pH scale. Wound margin is highlighted with a yellow dashed line. All scale bars 100 µm. **(B**) Linearized portion of a slice of the same pH map stack. (**C**) *XZ* maximum intensity projection. (**D**) pH as a function of distance from the wound. Images from B were averaged. Linear regression with 95% confidence level bands shows a significant increase (Deviation from slope zero test, P < 0.05). n = 6 mice.

The pH sensitive probe BCECF shows reduced emission intensity and lifetime in a protonated form. As reported^1^, BCECF FLIM images were fitted with a single exponential after subtraction of an estimated instrument response function (IRF). These images were then stitched to produce lifetime *XYZ* mosaics (600–1000 µm wide), from which we computed pH maps (Fig. 4A). To analyse the spatial distribution from the wound margin, the wound profile in the mosaics was then linearized (Fig. 4B) and pH was analysed as average in consecutive and equally distributed ROI bins from wound margin. *XZ* projections showed an acidic pH at the perilesional margins of 6.17 ± 0.58, which at 200 ± 35 μm switches to a more basic pH of 7.82 ± 0.24 (Fig. 4C). The pH curve showed a significant increase in pH as a function of distance in µm from the wound margin, with a positive slope of 0.0013 ± 0.00022 μm^−1^ when fitted with a linear regression model (Fig. 4D, darker green line with 95% CL bands. Deviation from zero slope test, P < 0.05). Therefore, results indicate a correlation between regions of increased NADH/NAD^+^ ratio as expression of augmented cell glycolytic activity ratio and hypoxia and acidification at wound margins.

### Measurement of cell glycolysis activity in cancer microenvironment by FLIM

**Figure 5:**
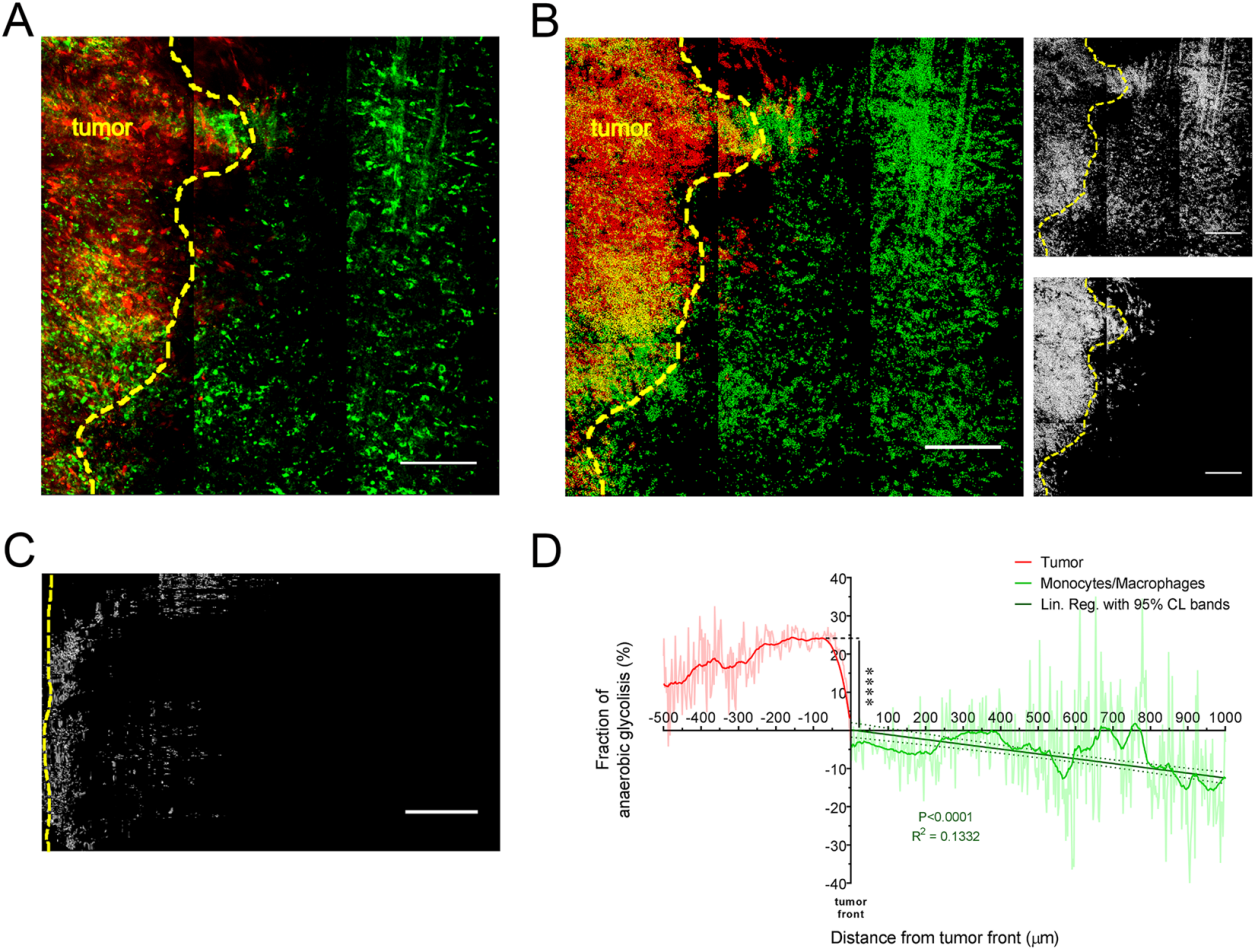
Measurement of anaerobic glycolysis in the tumour microenvironment by FLIM quantification of NADH/NAD^+^ ratio. CX3CR1^+/gfp^ mouse monocytes were i.d. injected 1-day prior acquisition. (**A**) Stack acquired with PMTs is used to prepare a binary mask of the two cell populations. Yellow dashed line highlights tumour front. All scale bars 200 µm. (**B**) Binary mask is applied to the NADH/NAD^+^ map to obtain a map subset for each cell population. (**C**) Representative portion of a straightened NADH/NAD^+^ ratio map for the GFP^+^ cells. (**D**) NADH/NAD^+^ ratio as a function of distance from the tumour front. Solid brighter lines were obtained by application of a LOWESS smoothing. Linear regression of the GFP^+^ cells (darkest green line with 95% confidence level bands) shows a decrease in NADH/NAD^+^ ratio in CX3CR1^+/gfp^ cells from the tumour front (deviation from slope zero test, P < 0.0001), while a Student *t*-test between the two populations shows that tumour cells globally have higher NADH/NAD^+^ ratio compared to the CX3CR1^+/gfp^ cells. ****P < 0.0001. n = 6 independent mice.

Solid tumours show an acidic microenvironment, which could be a determining factor in the induction of aggressive cancer phenotypes^20,26^. Metabolic adaptation in cancer is a key component of macrophage plasticity and definition of mechanisms regulating metabolic activity of macrophages, as well as the orchestration of metabolism by macrophages, are essential for the disease progression and represent a possible therapeutic intervention^10,23^. Measurement of NADH fluorescence lifetime was therefore extended in an experimental transplantable model of cancer in order to reconstruct a cellular metabolic map of macrophages in the tumour microenvironment (Fig. 5). mCherry-tagged cancer cell line CT26 were intradermally (i.d.) injected into CD-1 nude mice. On day 3, GFP^+^ monocytes isolated from the spleen of CX3CR1^+/gfp^ mice were i.d. injected in the peritumoral area. On day 4, dermis was surgically exposed with a skin flap and mounted into the imaging chamber with tumour at the centre. A first acquisition in standard imaging modality at 740 nm (Fig. 5A) was conducted to visualize the two cell populations and create their corresponding threshold-based binary masks. Thereafter, same *XYZ* area was then acquired in FLIM modality at 740 nm and analysed to obtain a NADH/NAD^+^ map of the tumour and peritumoral area. The two binary masks were then used to extract NADH/NAD^+^ maps of the corresponding cell populations (Fig. 5B). Images were then linearized from the tumour front to the peritumoral region for the GFP^+^ population (Fig. 5C), and from the tumour front to the inner region of the lesion for the mCherry^+^ population. As shown, at tumour-stromal interface mCherry^+^ and GFP^+^ cells showed a significantly higher NADH/NAD^+^ ratio corresponding to a higher level of anaerobic glycolysis compared to the surrounding tissue (P < 0.0001). At peritumoral region, linear regression (Fig. 5D, darker green line with 95% CL bands) of the NADH/NAD^+^ ratio associated with GFP^+^ cells showed a significant decrease (slope −0.0041 ± 0.00047 μm^−1^, deviation from zero slope test P < 0.0001).

## Discussion

Herein, we ascertained the effective relevance *in vivo* of cellular metabolic adaptation occurring in skin repair by interfering with anaerobic glycolytic activity. Moreover, we describe a protocol based on measurement of the intrinsic lifetime of NADH to reconstruct a spatial distribution of changes in anaerobic glycolysis correlated with areas of acidic pH and hypoxia in wound, as well as in cancer. The method was challenged by interfering with the anaerobic glycolysis through the use of DCA, an inhibitor of Pdk that leads to enhanced activity of pyruvate dehydrogenase thereby augmenting oxidative phosphorylation^44^. Moreover, we report evidence of modulation of the glycolytic gradient of the wound by interfering with the mitochondrial respiratory chain through the use of specific inhibitors.

In preclinical studies, the *in vivo* metabolic fluorescence imaging indirectly measures changes in oxygen tension, pH and glucose absorption in injured tissues and in tumour microenvironment by methods based on the detection of fluorescence intensity of specific sensitive probes, which suffer from local changes in probe concentrations and scattering. Ratiometric pH probes, such as seminaphtharhodafluor (SNARF), are intracellular pH indicators and are based on the intensity measurement at two emission wavelengths for the protonated and unprotonated forms of the dye^45^. Wavelength-dependent dyes can suffer from significant errors due to chromatic aberrations that can affect measurement especially in deeper portions of thick tissues^46^. Probes for optical *in vivo* imaging and positron-emission tomography (PET) exist to assess hypoxia^47^ and glucose^48^. Therefore, combination between optical *in vivo* imaging and specific fluorescent probes is suitable for quantifying the selected markers over a period of time, being also minimally invasive. However, it lacks appropriate spatial resolution: on a readily accessible organ such as the skin, the commonly achievable resolution ranges from 5 to 1 mm. Measurement of intrinsic lifetime of NADH and FAD is emerging as a reliable technique for assessing cellular metabolism parameters *in vitro*^40^. NADH FLIM also enabled analysis of pharmacologically-induced alterations to mitochondrial metabolic processes from baseline cerebral metabolism in rat cerebral cortex^37^ and metabolic changes at the front of wound re-epithelialization in diabetic and control mice^49^. This prompted us to apply protocol for measuring the spatial distribution of NADH in experimental models of tissue repair, as well as of cancer. Indeed, fluorescence decay time is an intrinsic quantity and quantification of FLIM is not subject to the same limitations as measurement of fluorescence intensity^34^. Moreover, 2PM excitation provides high penetration depth into the tissues for intravital imaging and cell resolution, allowing functional three-dimensional imaging.

In the clinic, the general method for the evaluation of skin pH is limited to measurements with electrodes^13,18^, which does not ensure adequate spatial resolution at the cellular level and a measurement limited to the most superficial part of the skin. Measurement of pH in non-healing or highly infected chronic wounds is an important element in the prediction of healing, as is the survival of skin graft in experimental and clinical studies and determines the success or failure of surgical treatment of burns and chronic ulcers^11,14,17^. Acidification of chronic venous ulcers is functionally relevant to the activities of remodelling cells, and therefore to their healing^11,16^. Overall, we describe a method that allows drawing a complete picture of wound metabolic phenotype and its spatial distribution, which could enhance the understanding of the relationship between microenvironment, metabolism and cell players in tissue repair. Moreover, this method could also represent a novel non-invasive diagnostic protocol for the monitoring of pathological wounds through the analysis of their glycolytic profile and the prediction of the outcome of surgical treatments.

## Methods

### Animals

C57Bl6/J female mice 8–14 weeks old (Charles River Laboratories) were used. 8–14 weeks old CX3CR1^+/gfp^ female mice were purchased from The Jackson Laboratory. Mice were housed in the specific pathogen–free animal facility of the Humanitas Clinical and Research Center in individually ventilated cages. Procedures involving animals handling and care were conformed to protocols approved by the Humanitas Clinical and Research Center (Rozzano, Milan, Italy) in compliance with national (4D.L. N.116, G.U., suppl. 40, 18–2-1992; D.L. N. 26, G.U. 4–3-2014) and international law and policies (EEC Council Directive 2010/63/EU, OJ L 276/33, 22–09-2010; National Institutes of Health Guide for the Care and Use of Laboratory Animals, US National Research Council, 2011, ARRIVE guidelines). The study was approved by the Italian Ministry of Health (approval n. 71/2012-B, issued on the 09/03/2012). All efforts were made to minimize the number of animals used and their suffering.

### Excisional model of skin injury

A full-thickness excisional skin injury model was performed as previously described^7^. Mice were anesthetized with 10 μL/g of weight of Ketamine (final concentration 10 mg/mL) and Xylazine (final concentration 1 mg/mL) in saline buffer. As described^7^, a full-thickness wound (from skin to underlying panniculus carnosus) was generated with disposable biopsy punch (8-mm diameter). Gentamicin was immediately applied to limit gross bacterial infections, not evident from histological examination carried out at different time points of the experiment. Mice were optionally treated with a solution of sodium dichloroacetate (DCA; Sigma-Aldrich Corp.), which was injected i.p. in 10–12 weeks-old C57BL/6J with a dosage of 300 mg/Kg 1 h before skin wounding. PBS or saline were used in control mice. As previously described^7^, the skin wounds of each mouse were digitally photographed and measured during wound healing by tracing them on a transparency, calculated with the ImageJ-Fiji software, and the variations in time expressed as a percentage of the initial wound area.

### Incisional model of skin injury

A full-thickness incisional skin injury model was performed with a scalpel in the centre region of the shaved back skin. We used C57Bl6/J mice of age between 8 and 14 weeks. Whole back skin was then hair-clipped and shaved with depilatory cream. Wound lesion was created by cutting the dorsal skin with a scalpel while pinching and pressing on a surface. The cut length was around 2–3 mm, resulting in a final wound size of about 4–6 mm. Mice olderthan 14 weeks showed more autofluorescence, which resulted in a longer time decay curve that could affect the exponential decay fitting in pH measurements and phasor analysis. DCA was used as described before. Rotenone (20 µM, 50 µl/mouse; Sigma-Aldrich Corp.) and Antimycin A (5 µM, 50 µl/mouse; Sigma-Aldrich Corp.) were finally resuspended in saline and i.d. injected around wound 1 h before imaging analyses.

### Transplantable model of cancer

Colon cancer cell line CT26 transfected with mCherry was prepared as described^50^ and i.d. injected in a CD-1 nude mouse (1 × 10^6^ cells in 100 μL). After 3 days, 7–8 × 10^5^ GFP^+^ monocytes were isolated from the spleen of CX3CR1^+/gfp^ mice and i.d. injected in the peritumoral area. Imaging analysis was conducted the day after their injection. The dermis was surgically exposed with a skin flap as previously reported^26^ and mounted with tumour at the centre of the imaging chamber’s window. A first acquisition in imaging modality at 740 nm was used to identify the two cell populations and create their corresponding threshold-based binary masks. The same *XYZ* area was then immediately acquired a second time in FLIM modality at 740 nm and analysed to obtain a global NADH/NAD^+^ map of the tumour and peritumoral area. The two binary masks were then used extract NADH/NAD^+^ maps of the corresponding cell populations. The NADH/NAD^+^ maps were then linearized and the intensity profile was measured.

### Intravital imaging

The implant procedure for the imaging chamber consists of pinching the dorsal skin on one block of the chamber after placing the mouse on one side, gently pressing the other block on the skin (Fig. S2). The pressure on the dorsal skin can be adjusted with the nuts, to avoid any restriction of blood flow. The imaging chamber is then mounted on a micrometre *XYZ* manipulator and lifted. The entire configuration creates a flat imaging window and greatly reduces motion artefacts due to the animal’s breathing. Animal temperature was kept at 37 °C with a heating pad (Fig. S2C), with mouse placed in lateral *decubitus*. Vital signs and anaesthesia level were regularly checked. Image mosaics were acquired with a 2-photon laser-scanning microscope (LaVision BioTec; Germany) in upright configuration for direct accessibility to the imaging window. Images were 200–250 μm wide, with a pixel size of 0.8 μm. The system is equipped with a Chameleon Ultra Ti:Sa laser (Coherent; USA), a 20X water-immersion objective (XLUMPLFLN-W, NA 1.0, Olympus;; Germany), two GaAsP photomultipliers (H6780-20) and a TCSPC x16 FLIM detector (LaVision BioTec; Germany). Emission filters for the imaging modality were 525/50 and 609/54, with a dichroic mirror at 593 nm.

### Measurement of glycolysis

Images were acquired in FLIM modality with excitation wavelength 740 nm and a power at sample range of 5 to 15 mW, with a pixel dwell of 5 to 15μs. 3D Mosaics were acquired with a tile overlap of 0% to avoid artefacts in photon counts at edges and Z step size of 5 to 10μm. We applied a 3×3 binning to the images to increase the signal-to-noise ratio, losing spatial resolution but gaining analysis accuracy, and then calculated the phasor plot with TCSPC Phasor Analysis Plugin for Fiji/ImageJ. Phasor plot was calibrated with fluorescein (1 mM; Sigma-Aldrich Corp.) solution (0.1 M NaOH), NADH (100 mM; Sigma-Aldrich Corp.) solution (0.1 M MOPS buffer, pH 7) and second harmonic signal (SHG) from urea crystals (Bio-Rad Laboratories; USA) (n = 5 measurements for both calibration points). We then designed an analysis tool that first divides the phasor cloud into five regions, corresponding to a gradient of prevailing anaerobic glycolysis in regions of lower lifetimes towards a region where more oxidative phosphorylation occurs^39^. To determine the number of divisions, we assumed that if there were a gradient, it would between a region of high anaerobic glycolysis and a flat region corresponding to the homeostatic condition, with an inflection point in the central region. To describe data with an inflection point at least four points are needed, but since the inflection point was likely to occur at the centre we took an odd number of bands, hence five was the minimum required. These bands were constructed from the major axis of the NADH phasor cloud^51^. Pixels belonging to each region are then associated to five discrete values of decreasing intensity, equally spaced from 0 to 100 (bins 0–20, 20–40, 40–60, 60–80, 80–100), starting with the highest values from anaerobic glycolysis. Quantification of pixel intensity is thus proportional to the relative increase in anaerobic glycolysis. Spatial distribution of this intensity from wound margin was then analysed with the mapping analysis described below. Values are expressed as difference from basal level of each mouse, as measured in a region distant from lesion margin.

### Measurement of hypoxia

Hypoxyprobe Pimonidazole-HCl (Pimo) (Hypoxyprobe; USA) was dissolved to a concentration of 100 mg/ml in PBS^++^, and 40 μL of Pimo were i.d. injected at perilesional area of the wound site. After 45 minutes, we injected 30 μL of anti-pimonidazole monoclonal antibody conjugated to Dylight 549 (Hypoxyprobe; USA) at 60 μg/ml. Images were acquired 45 minutes after the Ab injections in imaging modality, with an excitation wavelength of 780 nm and a power at sample of 3–5 mW. A line average of 2 was applied, for a total pixel dwell of 8 μs. 3D Mosaics were acquired with a tile overlap of 0% to avoid artefacts in photon counts at edges and Z step size of 5 to 10 μm. Images were then reconstructed with Fiji/ImageJ Stitching plugin and we measured the spatial distribution of fluorescence intensity with the mapping analysis described below.

### Measurement of pH

2′,7′-Bis-(2-Carboxyethyl)-5-(and-6)-Carboxyfluorescein (BCECF) (ThermoFisher Scientific; USA) was dissolved in PBS^++^ to a final stock concentration of 1.6 mM and then to a working solution of 80 μM in Ethanol 100%. 50 μL of the working solution were applied topically to portion of skin inside the imaging chamber 3 times, waiting for complete dye takeup between the applications. Images were acquired in FLIM modality with excitation wavelength 820 nm and a power at sample range from 5 to 15 mW. To reach at least 10^3^ photons per pixel, acquisition lines were summed to reach a pixel dwell range 30–45μs. 3D Mosaics were acquired with a tile overlap of 0% to avoid artefacts in photon counts at edges and Z step size of 5 to 10μm. To obtain single lifetime images, images were fitted with the FLIMfit software tool developed at Imperial College London, using a single exponential and 3 × 3 binning.

To compute pH from the fluorophore’s lifetime, we start from the Henderson-Hasselbach equation^1^

$$\text{pH}\,=\,\text{pKa}+\text{log}\,\frac{[{\text{BCECF}}^{-}]}{[\text{HBCECF}]}$$

Measured lifetime $\tau$ at a certain point can be expressed as weighted average of the lifetimes of protonated and unprotonated fluorophores, respectively $\text{high}$ and $\text{low}$

$$\tau =\frac{{n}_{high}{\tau }_{high}+{n}_{low}{\tau }_{low}}{{n}_{high}+{n}_{low}}$$

then

$$\frac{{n}_{high}}{{n}_{low}}=\frac{\tau -{\tau }_{low}}{{\tau }_{high}-\tau }$$

so that the Henderson-Hasselbach equation finally reads

$$\text{pH}\,=\,\text{pKa}+\log \frac{\tau -{\tau }_{low}}{{\tau }_{high}-\tau }$$

An analysis script employs this formula to compute corresponding pH from the lifetime images. Determination of the unprotonated and protonated lifetimes was extracted from a calibration curve that characterizes the lifetime distribution of the fluorophore at different pHs. As previously reported^1,7^, we corrected the lifetime with a multiplication factor of 0.9 to consider a different refractive index in tissue than solution. Contribution to the lifetime from Second Harmonic Generation of abundant skin collagen or autofluorescence may change the shorter or longer part of the fluorescence time decay. For these reasons we chose an excitation wavelength that minimizes both contributions, deconvolved the Instrument Response Function (IRF) measured from SHG-emitting samples and excluded longer times from the time decay window. Single pH images were then merged in a mosaic with Fiji/ImageJ Stitching plugin. Spatial distribution of pH was then analysed with the mapping analysis described below.

### Mapping analysis

The analysis of spatial distribution of glycolysis, pH and hypoxia were performed with a set of Fiji/ImageJ macros (Supplementary Data File 1). In the case of pH, the analysis first linearized the wound front and perilesional area, divided the linearized image in consecutive, equally spaced, ROI bins and measured the average values in each bin. For NADH/NAD^+^ maps and Pimo images, we first linearized the wound front and perilesional area and then measured the intensity profile.

### Statistical analysis

Data analyses and graph construction were performed using Prism 7 software (GraphPad; USA). The sample sizes for technical replicates are presented in the figure legends, and samples were randomly assigned to groups for experiments. Data are presented as means ± SEM unless otherwise stated. For analysis of the statistical significance by Student *t*-test, the data distribution in each experiment was checked for normality using the D’Agostino-Pearson test. P value for glycolytic index between tumour cells and GFP^+^ peritumoral cells was calculated using an independent-sample two-sided *t* test (Fig. 5D). Linear regression was calculated with a method of least squares, while deviation from slope test was used to assess the presence of a gradient while imposing the minimum set of assumptions on the profile function (Figs. 2D,E, 3D and 5D for the analysis of the fraction of anaerobic glycolysis in both tumour (x < 0) and peritumoral GFP^+^ cells (x > 0). Unless otherwise stated, in this study P < 0.05 was considered significant (*P < 0.05, **P < 0.01, ***P < 0.001, and ****P < 0.0001).

## Supplementary information

Supplementary material 1.

Supplementary material 2.

## Data Availability

All data needed to evaluate the conclusions in the paper are present in the paper and/or in the Supplementary Information. Additional data related to this paper may be requested to  the authors.
