# Author Correction: Evaluation of cell metabolic adaptation in wound and tumour by Fluorescence Lifetime Imaging Microscopy

**DOI:** 10.1038/s41598-020-71164-x

**Published:** 2020-08-19

**Authors:** Diego Morone, Francesca D’ Autilia, Tilo Schorn, Marco Erreni, Andrea Doni

**Affiliations:** 1 Unit of Advanced Optical Microscopy, IRCCS, Humanitas Clinical and Research Center, Rozzano, Milan Italy; 2 Present Address: Faculty of Biomedical Sciences, Institute for Research in Biomedicine, Università Della Svizzera Italiana (USI), Bellinzona, Switzerland

Correction to: *Scientific Reports* 10.1038/s41598-020-63203-4, published online 14 April 2020

This Article contains an error in the Acknowledgments section, where the funding source Fondazione CARIPLO (Contract no. 2015-0564 to Alberto Mantovani) was incorrectly added.

“We are grateful to the contribution of Giulia Maggi for the discussions on the analysis of the tumour glycolytic profile dataset. The financial supports of the Italian Association for Cancer Research (AIRC IG 23465; and AIRC 5 ×1000 21147 ISM), the Italian Ministry of Health and University (PRIN 2015YYKPNN to Alberto Mantovani) and Fondazione CARIPLO (Contract no. 2015-0564 to Alberto Mantovani) are gratefully acknowledged.”

should read:

“We are grateful to the contribution of Giulia Maggi for the discussions on the analysis of the tumour glycolytic profile dataset. The financial supports of the Italian Association for Cancer Research (AIRC IG 23465; and AIRC 5×1000 21147 ISM), the Italian Ministry of Health and University (PRIN 2015YYKPNN to Alberto Mantovani) are gratefully acknowledged.”

