# Supplementary material for: Evaluation of cell metabolic adaptation in wound and tumour by Fluorescence Lifetime Imaging Microscopy
Source: Sci Rep. 2020 Apr 14;10:6289. doi: 10.1038/s41598-020-63203-4 (PMC7156395; doi:10.1038/s41598-020-63203-4)
Supplement: Supplementary file 1 — Supplementary material 1. [file 41598_2020_63203_MOESM1_ESM.pdf]

SUPPLEMENTARY INFORMATION

Evaluation of cell metabolic adaptation in wound and tumour by Fluorescence Lifetime Imaging Microscopy

Diego Morone[1, †], Francesca D'Autilia[1], Tilo Schorn[1], Marco Erreni[1], Andrea Doni[1]*

[1], Unit of Advanced Optical Microscopy, IRCCS, Humanitas Clinical and Research Center, Rozzano, Milan, Italy

[†], current affiliation: Faculty of Biomedical Sciences, Institute for Research in Biomedicine, Università della Svizzera italiana (USI), Bellinzona, Switzerland

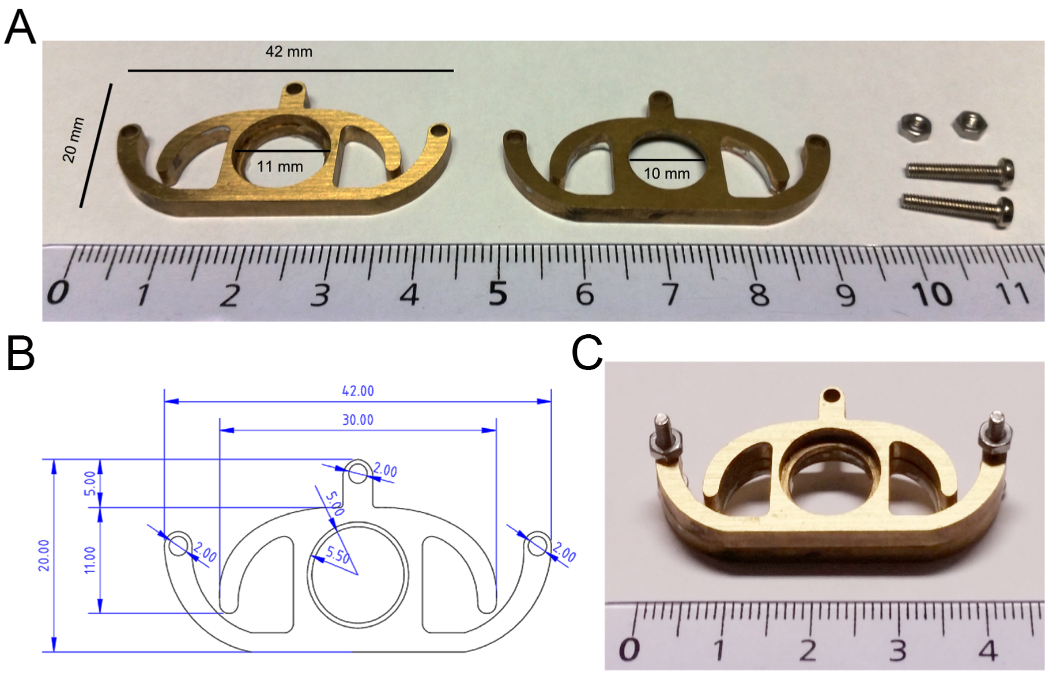

Supplementary Figure S1. View of skinfold chamber. A, the chamber shown from the exterior (left) and interior (right) part, with screws and bolts for mounting. The chamber has an imaging window of 10 mm. To further preserve the sample, a coverslip of size 11 mm can be mounted and fixed with an O-ring of same size. B, schematics of the chamber with dimensions. C, mounted chamber.

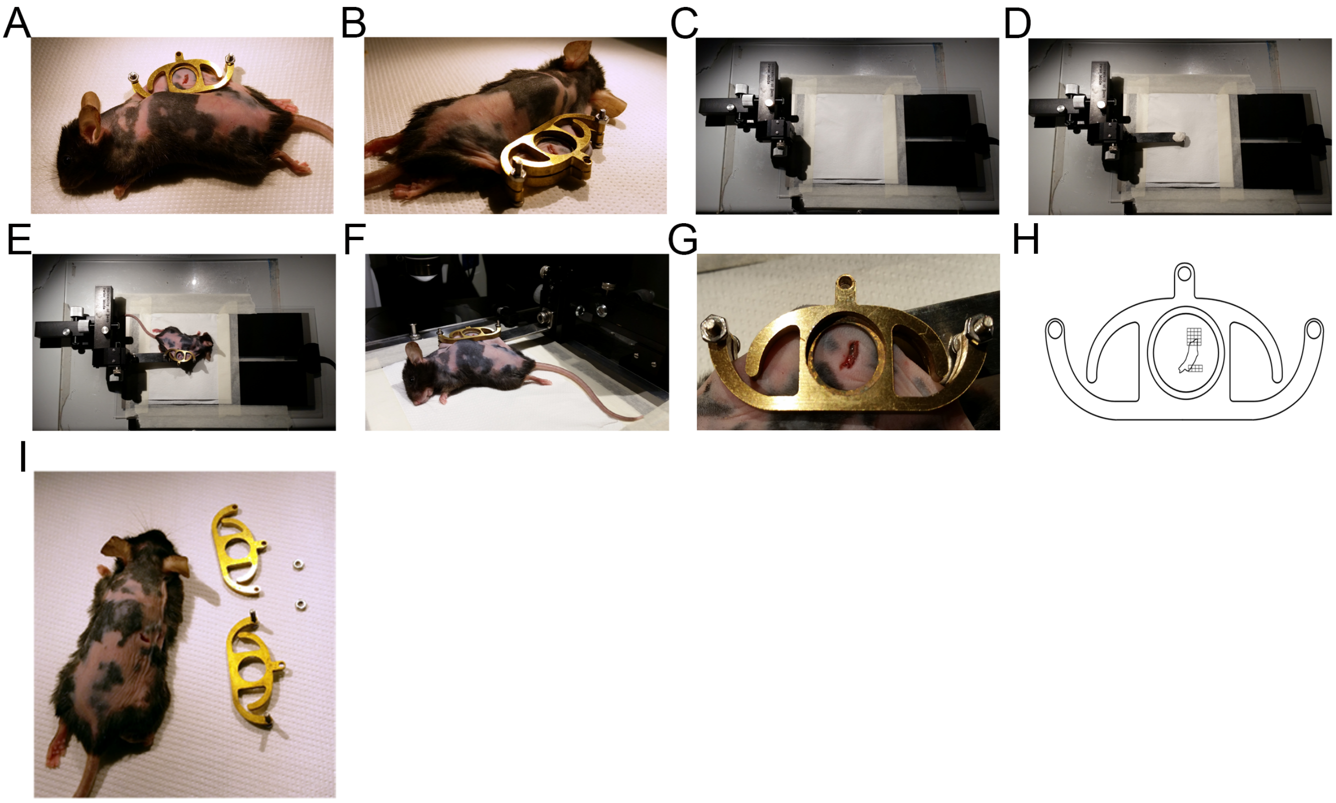

Supplementary Figure S2. Mouse preparation for 2P imaging. A, chamber is mounted on the mouse by pinching the back skin with forceps and enclosing the wound inside the chamber window. B, side view of the mounted chamber. C, intravital stage consists of a Plexiglas base, a thin heating pad, paper and a XYZ micrometric manipulator. D, L-shaped stainless-steel bracket is mounted on the XYZ manipulator. Skinfold chamber can be fixed on the bracket with plasticine or an additional screw. E, fixation of the skinfold chamber on the intravital stage. F, side view of the intravital stage on the microscope table. G, detail of the mounted imaging window. H, corresponding diagram with two possible imaging grids embracing wound bed and perilesional skin. I, skinfold chamber can be unmounted after imaging without damaging the skin.

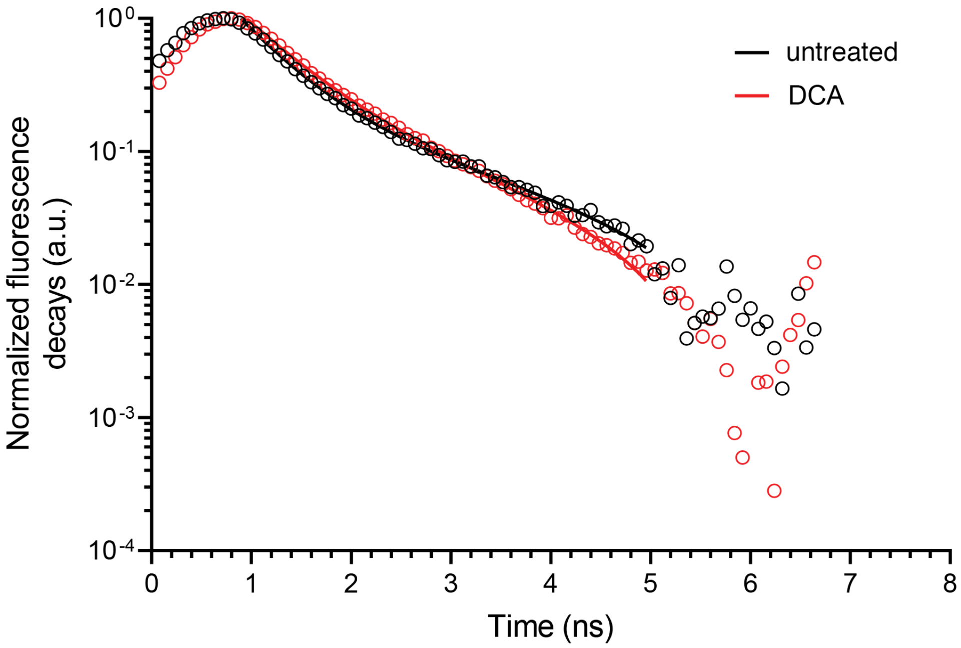

Two-phase exponential decay

untreated
Percentage of protein-bound NADH: 93.67 ± 0.74 %
Lifetimes: τ_1 = 2.69 ± 0.89 ns τ_2 = 0.48 ± 0.02 ns

DCA
Percentage of protein-bound NADH: 90.92 ± 3.03 %
Lifetimes: τ_1 = 2.05 ± 0.75 ns τ_2 = 0.54 ± 0.04 ns

Supplementary Figure S3. Two-phase exponential decay fit of NADH lifetime profile. Lifetime fit with a two-exponential decay curve of a perilesional region from single plane acquired with the same setting as Fig. 2 displays a short and long lifetime component, corresponding to free and proton-bound NADH, but fails to detect differences in the contributions. This approach is time consuming and can be misleading because free and protein-bound NADH have common exponential components and the NADH binding sites with different enzymes cannot considered.

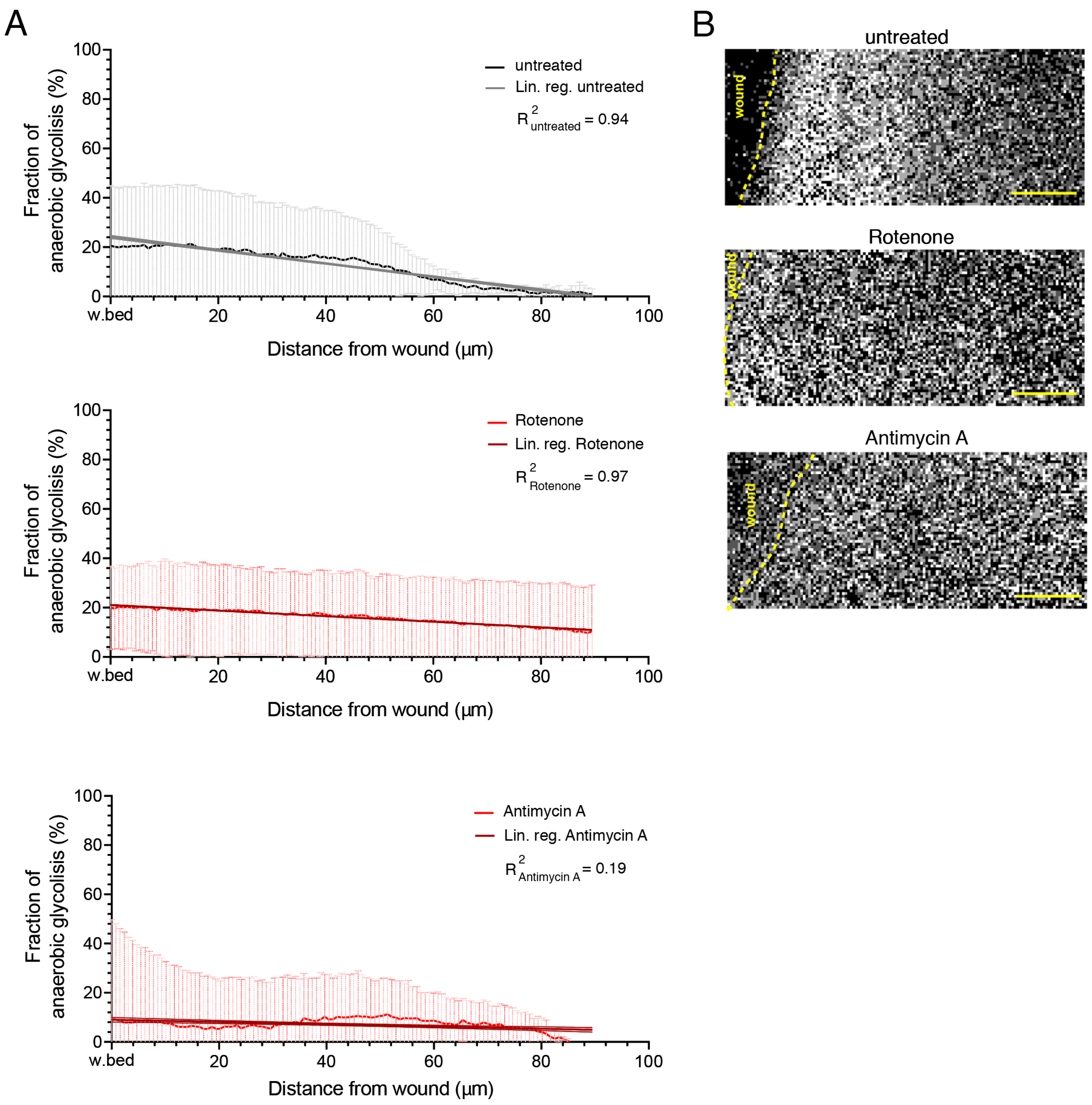

Figure S4. Effect of the mitochondrial respiratory chain in the measurement of NADH/NAD$^+$ ratio as expression of glycolysis in wound. A, NADH/NAD$^+$ ratio percentage as a function of distance from wound. Upon administration of Rotenone (red middle graph *vs* grey graph for untreated condition), NADH/NAD$^+$ ratio shows no significant decrease in value (comparison between first 10 values of each condition, *t*-test, P>0.05), but a significant reduction in line slope (Deviation between slopes test, P<0.0001. Deviation from zero slope test, P<0.0001). NADH/NAD$^+$ ratio is lower in perilesional area of Antimycin A-treated mice (red line in bottom graph *vs* grey line for untreated

condition, comparison between first 10 values of each condition, *t*-test, $P<0.0001$). Comparison with linear regressions with 95% confidence level bands (darker lines with bands) show a reduction in line slope in Antimycin A-treated mice (Deviation between slopes test, $P<0.0001$. Deviation from slope zero test, $P<0.0001$). B, map of a gradient of NADH/NAD+ ratio (grey) in wound in untreated (n=4) or mice treated with Rotenone (n=3) and Antimycin A (n=2). Wound margin is highlighted with a yellow dashed line. All scale bars 20μm.

Supplementary Data file 1. Macro file for image mapping analysis. Type: zip file containing a text file with ijm file extension, corresponding PDF with colour-highlighted syntax, a compiled ImageJ plugin file (.class), and its source code (.java file).
