# Supplementary material for: Evaluation of cell metabolic adaptation in wound and tumour by Fluorescence Lifetime Imaging Microscopy
Source: Sci Rep. 2020 Apr 14;10:6289. doi: 10.1038/s41598-020-63203-4 (PMC7156395; doi:10.1038/s41598-020-63203-4)
Supplement: Supplementary file 2 — Supplementary material 2. [file 41598_2020_63203_MOESM2_ESM.pdf]

```
/* 2017-04-04
 * Set of macros to stitch and analyze the FLIM data produced in Skin Wound
Healing experiments.
 *
 * This set of macros requires Fiji open source software (http://fiji.sc)
 * For the phasor analysis it also requires the TCPSC Time Gated Phasor
plugin
 * (http://www.spechron.com/Time%20gated%20Phasor-Download.aspx)
 *
 * To load in Fiji:
 *         Plugins>Macros>Install...
 *
 * You will then find all the macros at location
 *         Plugins>Macros
 *
 *
 * List of available macros
 * =======================
 *
 * Software Preparation
 * - Phasor Analysis Options
 * - pH Calibration Curve Limits...
 * - Stitching Preferences...
 * - Set Temp Directory...
 * - Set Profiles Directory...
 * - Log Preferences Values
 *
 * Phasor Analysis
 * - Stitch FLIM Data...
 * - Phasor (Shortcut: q)
 * - Phasor NAD+/NADH Ggradient (Shortcut: w)
 * - Save Gradient and Close (Shortcut: e)
 * - Merge and LUT for NAD+/NADH Gradient  (Shortcut: r)
 *
 * pH Analysis
 * - Stitch FLIMfit Data...
 * - Compute pH
 *
 * Pimonidazole Analysis
 * - Stitch PMT Data...
 *
 * Wound front linearization and analysis
 * - Straighten Profile  (Shortcut: t)
 * - Draw Half-Line Right (Shortcut: a)
 * - Draw Half-Line Left (Shortcut: s)
 * - Analyze NAD+/NADH Profile (Shortcut: d)
 * - Analyze pH Profile (Shortcut: f)
 * - Analyze Hypoxic Profile (Shortcut: g)
 */



/*
 * Set the preferences for phasor analyis
 */
macro "Phasor Analysis Options..." {
      Dialog.create("Phasor Analysis Options");
      Dialog.addNumber("Binning X", 3, 0, 6, "");
      Dialog.addNumber("Binning Y", 3, 0, 6, "" );
      Dialog.addNumber("Binning Lifetime", 1, 0, 6, "");
      Dialog.addNumber("Lifetime window", 10.96, 3, 6, "ns");
      Dialog.addNumber("Phasor image dimension", 400, 0, 6, "pixels");
      Dialog.addNumber("NAD-NADH gradient regions", 5, 0, 6, "");
      Dialog.show();
```

```
	binningX = round(Dialog.getNumber());
	binningY = round(Dialog.getNumber());
	binningZ = round(Dialog.getNumber());
	lifetimeWindow = Dialog.getNumber();
	phasorDim = round(Dialog.getNumber());
	nregions = round(Dialog.getNumber());
	call("ij.Prefs.set", "phasor.binningX", toString(binningX));
	call("ij.Prefs.set", "phasor.binningY", toString(binningY));
	call("ij.Prefs.set", "phasor.binningZ", toString(binningZ));
	call("ij.Prefs.set", "phasor.lifetimewindow",
toString(lifetimeWindow));
	call("ij.Prefs.set", "phasor.phasordim", toString(phasorDim));
	call("ij.Prefs.set", "phasor.nregions", toString(nregions));
}



/*
 * Set the calibration limits for BCECF dye
 */
macro "pH Calibration Curve Limits..." {
	tmphigh = call("ij.Prefs.get", "phmap.highlim", "2500");
	tmplow = call("ij.Prefs.get", "phmap.lowlim", "3600");
	tmpmult = call("ij.Prefs.get", "phmap.mult", "0.9");
	Dialog.create("pH Calibration Curve Limits");
	Dialog.addNumber("Low limit (ps):", tmplow, 0, 6, "");
	Dialog.addNumber("High limit (ps):", tmphigh, 0, 6, "" );
	Dialog.addNumber("Correction multiplication factor:", tmpmult, 0, 6, ""
);
	Dialog.show();
	lowlim = round(Dialog.getNumber());
	highlim = round(Dialog.getNumber());
	mult = round(Dialog.getNumber());
	call("ij.Prefs.set", "phmap.lowlim", toString(lowlim));
	call("ij.Prefs.set", "phmap.highlim", toString(highlim));
	call("ij.Prefs.set", "phmap.mult", toString(mult));
}



/*
 * Set the image rotation preferences for image stitching.
 * Depending on the acquisition setup and on the software, you may need to
enable
 * some of these options to properly align the images for stitching.
 */
macro "Stitching Preferences..." {
	Dialog.create("Stitching preferences");
	Dialog.addCheckbox("Rotate 90 degrees left", false);
	Dialog.addCheckbox("Flip", false);
	Dialog.show();
	rotate = Dialog.getCheckbox();
	flip = Dialog.getCheckbox();
	call("ij.Prefs.set", "stitching.rotate", rotate);
	call("ij.Prefs.set", "stitching.flip", flip);
}



/*
 * Temporary directory where images for each slice will be saved before
stitching.
 * This should be a local directory to increase stitching performance
 */
```

```
macro "Set Temp Directory..." {
      newtmpdir = getDirectory("Set path for new tempdir on LOCAL path");
      call("ij.Prefs.set", "stitching.tmpdir",toString(newtmpdir));
}



/*
 * Temporary directory where straighten profiles for each slice will be
saved.
 * Remember to copy these images to a new folder before continuing with the
next image.
 */
macro "Set Profiles Directory..." {
      newtmpdir = getDirectory("Set path for new plot profiles dir on LOCAL
path");
      call("ij.Prefs.set", "phasor.plotprofilesdir",toString(newtmpdir));
}



/*
 * Create a log window of all preferences
 */
macro "Log Preferences Values" {
      IJ.log("--- BEGIN Preferences ---");
      IJ.log("--- Phasor Preferences ---");
      IJ.log("Binning X: "+call("ij.Prefs.get", "phasor.binningX", "Not
set"));
      IJ.log("Binning Y: "+call("ij.Prefs.get", "phasor.binningY", "Not
set"));
      IJ.log("Binning Z: "+call("ij.Prefs.get", "phasor.binningZ", "Not
set"));
      IJ.log("Phasor Lifetime Window (ns): "+call("ij.Prefs.get",
"phasor.lifetimewindow", "Not set"));
      IJ.log("Phasor Image Dimension (pixels): "+call("ij.Prefs.get",
"phasor.phasordim", "Not set"));
      IJ.log("Number of phasor regions: "+call("ij.Prefs.get",
"phasor.nregions", "Not set"));
      IJ.log("--- Stitching Preprocessing Preferences ---");
      IJ.log("Rotate FLIM images: "+call("ij.Prefs.get",
"stitching.rotate","Not set"));
      IJ.log("Flip FLIM images: "+call("ij.Prefs.get", "stitching.flip","Not
set"));
      IJ.log("--- Folder preferences ---");
      IJ.log("Temp images: "+call("ij.Prefs.get", "stitching.tmpdir","Not
set"));
      IJ.log("Phasor Profiles: "+call("ij.Prefs.get",
"phasor.plotprofilesdir","Not set"));
      IJ.log("--- pH Calibration values ---");
      IJ.log("Lower Limit (ps): "+call("ij.Prefs.get", "phmap.lowlim", "Not
set"));
      IJ.log("Higher Limit (ps): "+call("ij.Prefs.get", "phmap.highlim", "Not
set"));
      IJ.log("--- END Preferences ---");
}



macro "-" {} //menu divider
```

```
/*
 * This macro stitches FLIM images. Each image is a z-stack, containing TCPSC
data
 * for a single position in the stitching volume.
 *
 */
macro "Stitch FLIM Data..." {
	setBatchMode(true);
	var stitchO = "0";
	var dataindicator="TDC";
	indir = getDirectory("input dir");
	outdir = substring(indir, 0, lengthOf(indir) -1) + " stitch\\";
	intensitydir = substring(indir, 0, lengthOf(indir) -1) + "
intstitch\\";

	var tmpdir=call("ij.Prefs.get", "stitching.tmpdir","");
	if (tmpdir == "") {
		Dialog.create("Cache Folder does not exist");
		Dialog.addMessage("Need to define a cache folder!");
		Dialog.show();
		tmpdir = getDirectory("Select a Cache Folder");
		call("ij.Prefs.set", "stitching.tmpdir", tmpdir);
		exit("Restart Macro to go");
	}

	var filebase = "", stitchX =0, stitchY=0, stitchZ=0, stitchC=0,
pxx="1.000000", pxy="1.000000", pxz="1.000000";
	var is2d = 0;

	if (!File.exists(tmpdir)) {
		File.makeDirectory(tmpdir);
	} else {
		tmplist=getFileList(tmpdir);
		var ok = 1;
		for (i=0; i<tmplist.length; i++) {
			ok = File.delete(tmpdir+tmplist[i]);
			if (ok == 0)
				IJ.log("Cannot delete temp file"+tmpdir+tmplist[i]);
		}
	}
	if (!File.exists(outdir))
		File.makeDirectory(outdir);
	if (!File.exists(intensitydir))
		File.makeDirectory(intensitydir);

	list = getFileList(indir);
	filebaseindex=0;
	do {
		filebaseindex++;
	} while (!matches(list[filebaseindex], ".*[0-9]{2}-[0-9]{2}-[0-
9]{2}_.*_C[0-9]{2}.*\\.ome\\.tif"));
	filebase = substring(list[filebaseindex], 0,
indexOf(list[filebaseindex],dataindicator));

	// Confirmation dialog before starting
	Dialog.create("Stitching properties confirmation");
	Dialog.addNumber("Calibration X (um)", pxx);
	Dialog.addNumber("Calibration Y (um)", pxy);
	Dialog.addNumber("Calibrazion Z (um)", pxz);
	Dialog.show();
	pxx = Dialog.getNumber();
	pxy = Dialog.getNumber();
	pxz = Dialog.getNumber();
```

```
	for (i= 0; i< list.length; i++) {
		if (matches(list[i], ".*[0-9]{2}-[0-9]{2}-[0-9]{2}_.*_C[0-9]{2}.*Table Z.*\\.ome\\.tif")) {
			tmp = parseInt(substring(list[i],indexOf(list[i],"[")+1,indexOf(list[i]," x")));
			stitchY = maxOf(stitchY, tmp);

			tmp = parseInt(substring(list[i],indexOf(list[i]," x ")+3,indexOf(list[i],"]")));
			stitchX = maxOf(stitchX, tmp);

			tmp = parseInt(substring(list[i],indexOf(list[i],"Table Z")+7,indexOf(list[i],".ome")));
			stitchZ = maxOf(stitchZ, tmp);

			tmp = parseInt(substring(list[i],indexOf(list[i],"_C")+2,indexOf(list[i],"_xyz-Table")));
			stitchC = maxOf(stitchC, tmp);
		} else if (matches(list[i], ".*[0-9]{2}-[0-9]{2}-[0-9]{2}_.*_C[0-9]{2}\\.ome\\.tif")) {
			is2d =1;

			tmp = parseInt(substring(list[i],indexOf(list[i],"[")+1,indexOf(list[i]," x")));
			stitchY = maxOf(stitchY, tmp);

			tmp = parseInt(substring(list[i],indexOf(list[i]," x ")+3,indexOf(list[i],"]")));
			stitchX = maxOf(stitchX, tmp);

			tmp = parseInt(substring(list[i],indexOf(list[i],"_C")+2,indexOf(list[i],".ome")));
			stitchC = maxOf(stitchC, tmp);
		}
	}
	stitchY=stitchY+1;
	stitchX=stitchX+1;
	IJ.log(stitchY+","+stitchX+","+stitchC+","+stitchZ);

	if (is2d == 1) {
		var stitchrotate = call("ij.Prefs.get", "stitching.rotate","");
		var stitchflip = call("ij.Prefs.get", "stitching.flip","");
		for (i=0; i< list.length; i++) {
			if (matches(list[i], filebase + ".*\\.ome\\.tif")) {
				open(indir + list[i]);

				if (stitchrotate) {run("Rotate 90 Degrees Left")};
				if (stitchflip) {run("Flip Horizontally", "stack")};

				nobrackets = replace(list[i], "\\[(.*?)\\]", "$1");
				saveAs("TIFF", tmpdir + nobrackets);
			}
		}

		for (c=0; c <= stitchC; c++) {
			run("Grid/Collection stitching", "type=[Filename defined position] order=[Defined by filename         ] grid_size_x="+stitchX+" grid_size_y="+stitchY+" tile_overlap="+stitchO+" first_file_index_x=0 first_file_index_y=0 directory="+tmpdir+" file_names=["+filebase+dataindicator+"{yy} x {xx}_C"+IJ.pad(c,2)+".ome.tif] output_textfile_name=TileConfiguration.txt fusion_method=[Max. Intensity] regression_threshold=0.30 max/avg_displacement_threshold=2.50
```

```
absolute_displacement_threshold=3.50 subpixel_accuracy
computation_parameters=[Save computation time (but use more RAM)]
image_output=[Fuse and display]");
				run("Properties...", "channels=1 slices=1 frames=1 unit=um
pixel_width="+pxx+" pixel_height="+pxy+" voxel_depth="+pxz);
				saveAs("TIFF", outdir + filebase + "C" + IJ.pad(c,2) +
".tif");
			}
			tmplist=getFileList(tmpdir);
			var ok = 1;
			for (i=0; i<tmplist.length; i++) {
				ok = File.delete(tmpdir+tmplist[i]);
				if (ok == 0)
					IJ.log("Cannot delete temp file"+tmpdir+tmplist[i]);
			}

		} else  {
			for (z=0; z <= stitchZ; z++) {
				for (i=0; i< list.length; i++) {
					if (matches(list[i], filebase + ".*Table Z" +
IJ.pad(z,4) + ".*\\.ome\\.tif")) {
						open(indir + list[i]);

						stitchrotate = call("ij.Prefs.get",
"stitching.rotate","");
						stitchflip = call("ij.Prefs.get",
"stitching.flip","");
						if (stitchrotate) {run("Rotate 90 Degrees
Left")};
						if (stitchflip) {run("Flip Horizontally",
"stack")};

						nobrackets = replace(list[i], "\\[(.*?)\\]",
"$1");
						saveAs("TIFF", tmpdir + nobrackets);
					}
				}

				for (c=0; c <= stitchC; c++) {
					run("Grid/Collection stitching", "type=[Filename
defined position] order=[Defined by filename         ]
grid_size_x="+stitchX+" grid_size_y="+stitchY+" tile_overlap="+stitchO+"
first_file_index_x=0 first_file_index_y=0 directory="+tmpdir+"
file_names=["+filebase+dataindicator+"{yy} x {xx}_C"+IJ.pad(c,2)+"_xyz-Table
Z"+IJ.pad(z,4)+".ome.tif] output_textfile_name=TileConfiguration.txt
fusion_method=[Linear Blending] regression_threshold=0.30
max/avg_displacement_threshold=2.50 absolute_displacement_threshold=3.50
subpixel_accuracy computation_parameters=[Save computation time (but use more
RAM)] image_output=[Fuse and display]");
					run("Properties...", "channels=1 slices=1 frames=1
unit=um pixel_width="+pxx+" pixel_height="+pxy+" voxel_depth="+pxz);
					saveAs("TIFF", outdir + filebase + "C" + IJ.pad(c,2)
+ "_Z" + IJ.pad(z,4) + ".tif");
				}
				tmplist=getFileList(tmpdir);
				var ok = 1;
				for (i=0; i<tmplist.length; i++) {
					ok = File.delete(tmpdir+tmplist[i]);
					if (ok == 0)
						IJ.log("Cannot delete temp
file"+tmpdir+tmplist[i]);
				}
			}
		}
```

```
      flimlist = getFileList(outdir);
      for (i=0; i<flimlist.length; i++) {
            if (matches(flimlist[i], ".*_C[0-9]{2}_Z[0-9]{4}\\.tif")) {
                  open(outdir + flimlist[i]);
                  run("Z Project...", "projection=[Sum Slices]");
                  saveAs("TIFF", intensitydir + flimlist[i]);
            }
      }
      setBatchMode(false);
      IJ.log("====== "+filebase+"FLIM Data Stitching Completed. ======");

}



/*
 * Recall preferences for phasor plugin and run the plugin with the currently
open image
 */
var phasorimageID; // stores the ID of the image from which the phasor is
calculated. Useful for macro "Save gradient and close"
macro "Phasor[q]" {
      phasorimageID = getImageID();
      var binningX=call("ij.Prefs.get", "phasor.binningX","");
      var binningY=call("ij.Prefs.get", "phasor.binningY","");
      var binningZ=call("ij.Prefs.get", "phasor.binningZ","");
      var lifetimeWindow=call("ij.Prefs.get", "phasor.lifetimewindow","");
      var phasorDim=call("ij.Prefs.get", "phasor.phasordim","");
      run("Bin...", "x="+binningX+" y="+binningX+" z="+binningZ+" bin=Sum");
      run("Timegatedphasor ", "threshold=0 total="+lifetimeWindow+" back=0
phasor="+phasorDim+" reference=3.98 options=[Pixel by Pixel]");
      run("ROI Manager...");
      // select regions to extract
}



/*
 * This macro divides the phasor diagram into a specified number of regions,
and returns a stack
 * where each image corresponds to each of these regions.
 * To run, draw a straight line thru the phasor diagram region as discussed
in the text, then
 * select the desired region size with Edit>Options>Line Width... and run
macro
 */
macro "Phasor NAD+/NADH Gradient[w]" {
      var binningX=call("ij.Prefs.get", "phasor.binningX","");
      var binningY=call("ij.Prefs.get", "phasor.binningY","");
      var binningZ=call("ij.Prefs.get", "phasor.binningZ","");
      var lifetimeWindow=call("ij.Prefs.get", "phasor.lifetimewindow","");
      var phasorDim=call("ij.Prefs.get", "phasor.phasordim","");
      var nregions=call("ij.Prefs.get", "phasor.nregions","");
      /* NB Signs are a bit odd to take into account that the origin
       *    is the upper left corner:
       *    y axis = -y
       */
      title=getTitle();
      getLine(x1, y1, x2, y2, lineWidth);
      if (lineWidth <= 0)
            exit("Please provide a line width (Edit->Selection->Properties...
or Ctrl+Y)");
      linelength = sqrt(pow(abs(x1-x2),2)+pow(abs(y1-y2),2));
      radangle = atan2(y1-y2,x2-x1);
```

```
	reglength = round(linelength/nregions);
	roiManager("Reset");
	for ( i = 0 ; i < nregions; i++) {
		xtmp1 = x1+i*reglength*cos(radangle);
		xtmp2 = x1+(i+1)*reglength*cos(radangle);
		ytmp1 = y1-i*reglength*sin(radangle);
		ytmp2 = y1-(i+1)*reglength*sin(radangle);
		makeLine(xtmp1,ytmp1,xtmp2,ytmp2,lineWidth);
		run("Line to Area");
		roiManager("Add");
	}
	n = roiManager("count");
	for (i=0; i<n; i++) {
		selectWindow(title);
		roiManager("select", i);
		run("Phasor To Image");
		rename("nadh-gradient"+(i+1));
		selectWindow("Spectrum");
		run("Close");
	}
	run("Merge Channels...", "c1=nadh-gradient1 c2=nadh-gradient2 c3=nadh-
gradient3 c4=nadh-gradient4 c5=nadh-gradient5 create");
}



/*
 *  This command keeps the original image open and closes all other
 *  open images. To use after saving the image gradient generated before
 *  moving to next image to be analyzed.
 */
macro "Save Gradient and Close[e]" {
	currentImageID=phasorimageID;		//get image ID for current
image
	totalOpenImages=nImages;		//get total number of open
images
	imageIDs=newArray(nImages);		//create array to hold all image
IDs
	for(i=0;i<nImages;i++){				//and populate array
with image IDs
		selectImage(i+1);
		imageIDs[i]=getImageID();
	}

	for(i=0;i<totalOpenImages;i++){		//run through array of image
IDs
		if(imageIDs[i]!=currentImageID){	//and check whether it
matches current image
			selectImage(imageIDs[i]);		//if it doesn't
match,select image and close it
			close();
		}
	}
}



/*
 * Combine the NADH gradient stack into a single image, where the
 */
macro "Merge and LUT for NAD+/NADH Gradient[r]" {
	var nregions=call("ij.Prefs.get", "phasor.nregions","");
	title=getTitle();
	getDimensions(w,h,c,s,dummy);
```

```
	run("Split Channels");
	newImage("Gradient", "8-bit black",w,h,s);
	for(i=1; i<=c; i++) {
		selectWindow("C"+i+"-"+title);
		run("8-bit");
		run("Multiply...", "value=1000000 stack");
		run("Divide...", "value=255 stack");

		multiplier = floor((1-(i-1)/nregions)*100);
		run("Multiply...", "value="+multiplier+" stack");
		imageCalculator("Add stack", "Gradient","C"+i+"-"+title);
	}
	selectWindow("Gradient");
}



macro "-" {} //menu divider



/*
 * This macro stitches together lifetime images fitted with FLIMfit
 */
macro "Stitch FLIMfit Data..." {
	setBatchMode(true);
	var stitchO = "0";
	var dataindicator="TDC";
	var indir = getDirectory("input dir");
	var outdir = substring(indir, 0, lengthOf(indir) -1) + " stitch\\";

	var tmpdir=call("ij.Prefs.get", "stitching.tmpdir","");
	if (tmpdir == "") {
		Dialog.create("Cache Folder does not exist");
		Dialog.addMessage("Need to define a cache folder!");
		Dialog.show();
		tmpdir = getDirectory("Select a Cache Folder");
		call("ij.Prefs.set", "stitching.tmpdir", tmpdir);
		exit("Restart Macro to go");
	}

	var filebase = "", stitchX =0, stitchY=0, stitchZ=0, stitchC=0,
pxx="1.000000", pxy="1.000000", pxz="1.000000";

	if (!File.exists(tmpdir)) {
		File.makeDirectory(tmpdir);
	} else {
		tmplist=getFileList(tmpdir);
		var ok = 1;
		for (i=0; i<tmplist.length; i++) {
			ok = File.delete(tmpdir+tmplist[i]);
			if (ok == 0)
				IJ.log("Cannot delete temp file"+tmpdir+tmplist[i]);
		}
	}
	if (!File.exists(outdir))
		File.makeDirectory(outdir);

	list = getFileList(indir);
	filebaseindex=0;
	do {
		filebaseindex++;
	} while (!matches(list[filebaseindex], ".*[0-9]{2}-[0-9]{2}-[0-
9]{2}_.*_C[0-9]{2}.*raw\\.tiff"));
```

```
	filebase = substring(list[filebaseindex], 0,
indexOf(list[filebaseindex],dataindicator));

	open(list[filebaseindex]);
	getDimensions(imsize, dummy, dummy, dummy, dummt);

	// Confirmation dialog before starting
	Dialog.create("Stitching properties confirmation");
	Dialog.addNumber("Calibration X (um)", pxx);
	Dialog.addNumber("Calibration Y (um)", pxy);
	Dialog.addNumber("Calibrazion Z (um)", pxz);
	Dialog.show();
	pxx = Dialog.getNumber();
	pxy = Dialog.getNumber();
	pxz = Dialog.getNumber();


	for (i= 0; i< list.length; i++) {
		if (matches(list[i], ".*[0-9]{2}-[0-9]{2}-[0-9]{2}_.*_C[0-
9]{2}.*raw\\.tiff")) {
			tmp =
parseInt(substring(list[i],indexOf(list[i],"[")+1,indexOf(list[i]," x")));
			stitchY = maxOf(stitchY, tmp);

			tmp = parseInt(substring(list[i],indexOf(list[i]," x
")+3,indexOf(list[i],"]")));
			stitchX = maxOf(stitchX, tmp);

			tmp = parseInt(substring(list[i],indexOf(list[i],"Table
Z")+7,indexOf(list[i],".ome")));
			stitchZ = maxOf(stitchZ, tmp);

			tmp =
parseInt(substring(list[i],indexOf(list[i],"_C")+2,indexOf(list[i],"_xyz-
Table")));
			stitchC = maxOf(stitchC, tmp);

			tmp =
parseInt(substring(list[i],indexOf(list[i],"tau_")+4,indexOf(list[i],"
raw")));
			stitchTau = maxOf(stitchTau, tmp);
		}
	}
	stitchY=stitchY+1;
	stitchX=stitchX+1;
	IJ.log(stitchY+","+stitchX+","+stitchC+","+stitchZ);

	for (f =1; f<= stitchTau; f++) {
		for (z=0; z <= stitchZ; z++) {
			for (i=0; i< list.length; i++) {
				if (matches(list[i], filebase + ".*Table Z" +
IJ.pad(z,4) + ".*raw\\.tiff")) {
					nobrackets = replace(list[i],
".*\\[(.*)\\](.*)xyz-Table (.*)\\.ome tau_"+f+" raw\\.tiff", "$1$2$3.tif");
					open(indir + list[i]);
					title=getTitle();
					//run("Rotate 90 Degrees Left");
					//run("Flip Horizontally", "stack");
					selectWindow(title);
					saveAs("TIFF", tmpdir + nobrackets);
				}
			}
			for (c=0; c <= stitchC; c++) {
				run("Grid/Collection stitching", "type=[Filename
```

```
defined position] order=[Defined by filename         ]
grid_size_x="+stitchX+" grid_size_y="+stitchY+" tile_overlap="+stitchO+"
first_file_index_x=0 first_file_index_y=0 directory="+tmpdir+"
file_names=[{yy} x {xx}_C"+IJ.pad(c,2)+"_Z"+IJ.pad(z,4)+".tif]
output_textfile_name=TileConfiguration.txt fusion_method=[Max. Intensity]
regression_threshold=0.30 max/avg_displacement_threshold=2.50
absolute_displacement_threshold=3.50 subpixel_accuracy
computation_parameters=[Save computation time (but use more RAM)]
image_output=[Fuse and display]");
                        run("Properties...", "channels=1 slices=1 frames=1
unit=um pixel_width="+pxx+" pixel_height="+pxy+" voxel_depth="+pxz);
                        saveAs("TIFF", outdir + filebase + "C" + IJ.pad(c,2)
+ "_Z" + IJ.pad(z,4) + "tau_"+f+".tif");
                  }
                  tmplist=getFileList(tmpdir);
                  var ok = 1;
                  for (i=0; i<tmplist.length; i++) {
                        ok = File.delete(tmpdir+tmplist[i]);
                        if (ok == 0)
                              IJ.log("Cannot delete temp
file"+tmpdir+tmplist[i]);
                  }
            }
      }
      setBatchMode(false);
      IJ.log("====== "+filebase+"FLIMfit Data Stitching Completed. ======");
}



/*
 * This macro takes either single lifetime images or stitched ones and
creates a pH map
 */
macro "Compute pH" {
      var lowlim = call("ij.Prefs.get", "phmap.lowlim", "2507");
      var hilim = call("ij.Prefs.get", "phmap.highlim", "3566");
      var mult = call("ij.Prefs.get", "phmap.mult", "0.9");
      title = getTitle();
      getDimensions(w,h,channels,slice,frame);
      run("32-bit");


      for (c=1; c <= channels; c++) {
            Stack.setChannel(c);
            for (z=1; z <= slice; z++) {
                  setSlice(z);
                  for (x=0 ; x < w ; x++) {
                        for (y=0; y < w; y++) {
                              tmp = getPixel(x,y);
                              setPixel(x,y,compute_ph(tmp));
                        }
                  }
            }
      }

      setMinAndMax(4,10);
      run("Rainbow RGB");

      function compute_ph(value) {
            value = mult * value;
            if (value > lowlim && value < hilim) {
                  return 7.0 + log((value - lowlim)/(hilim - value))
            } else
```

```
                  return 0
      }
}


macro "-" { } //menu divider


/*
 * Stitch images acquired in PMT modality (i.e. Pimo dye channel)
 */
macro "Stitch PMT Data..." {
      setBatchMode(true);
      var stitchO = "0";
      var dataindicator="PMT";
      indir = getDirectory("input dir");
      outdir = substring(indir, 0, lengthOf(indir) -1) + "_stitch\\";
      var tmpdir=call("ij.Prefs.get", "stitching.tmpdir","");
      var filebase = "", stitchX =0, stitchY=0, stitchZ=0, stitchC=0,
pxx="1.000000", pxy="1.000000", pxz="1.000000";

      if (!File.exists(tmpdir)) {
            File.makeDirectory(tmpdir);
      } else {
            tmplist=getFileList(tmpdir);
            var ok = 1;
            for (i=0; i<tmplist.length; i++) {
                  ok = File.delete(tmpdir+tmplist[i]);
                  if (ok == 0)
                        IJ.log("Cannot delete temp file"+tmpdir+tmplist[i]);
            }
      }
      if (!File.exists(outdir))
            File.makeDirectory(outdir);

      list = getFileList(indir);
      filebaseindex=0;
      do {
            filebaseindex++;
      } while (!matches(list[filebaseindex], ".*[0-9]{2}-[0-9]{2}-[0-
9]{2}_.*_C[0-9]{2}.*\\.ome\\.tif"));
      filebase = substring(list[filebaseindex], 0,
indexOf(list[filebaseindex],dataindicator));

      open(list[filebaseindex]);
      getDimensions(imsize, dummy, dummy, dummy, dummt);

      // Confirmation dialog before starting
      Dialog.create("Stitching properties confirmation");
      Dialog.addNumber("Calibration X (um)", pxx);
      Dialog.addNumber("Calibration Y (um)", pxy);
      Dialog.addNumber("Calibrazion Z (um)", pxz);
      Dialog.show();
      pxx = Dialog.getNumber();
      pxy = Dialog.getNumber();
      pxz = Dialog.getNumber();


      for (i= 0; i< list.length; i++) {
            if (matches(list[i], ".*[0-9]{2}-[0-9]{2}-[0-9]{2}_.*_C[0-
9]{2}.*\\.ome\\.tif")) {
                  tmp =
```

```
parseInt(substring(list[i],indexOf(list[i],"[")+1,indexOf(list[i]," x")));
			stitchY = maxOf(stitchY, tmp);

			tmp = parseInt(substring(list[i],indexOf(list[i]," x
")+3,indexOf(list[i],"]")));
			stitchX = maxOf(stitchX, tmp);

			tmp = parseInt(substring(list[i],indexOf(list[i],"Table
Z")+7,indexOf(list[i],".ome")));
			stitchZ = maxOf(stitchZ, tmp);

			tmp =
parseInt(substring(list[i],indexOf(list[i],"_C")+2,indexOf(list[i],"_xyz-
Table")));
			stitchC = maxOf(stitchC, tmp);
		}
	}
	stitchY=stitchY+1;
	stitchX=stitchX+1;
	IJ.log(stitchY+","+stitchX+","+stitchC+","+stitchZ);
	pmtnames = newArray(stitchC+1);
	for (i = 0 ; i< list.length; i++) {
		if (matches(list[i], ".*[0-9]{2}-[0-9]{2}-[0-9]{2}_.*_C[0-
9]{2}.*\\.ome\\.tif")) {
			tmp =
parseInt(substring(list[i],indexOf(list[i],"_C")+2,indexOf(list[i],"_xyz-
Table")));
			pmtnames[tmp] =
substring(list[i],indexOf(list[i],"_PMT")+4,indexOf(list[i],"["));
		}
	}
	for (i = 0 ; i< pmtnames.length; i++) {
		IJ.log(pmtnames[i]);
	}


	for (z=0; z <= stitchZ; z++) {
		for (i=0; i< list.length; i++) {
			if (matches(list[i], filebase + ".*Table Z" + IJ.pad(z,4) +
".*\\.ome\\.tif")) {
				open(indir + list[i]);
				nobrackets = replace(list[i], "\\[(.*?)\\]", "$1");
				saveAs("TIFF", tmpdir + nobrackets);
			}
		}

		for (c=0; c <= stitchC; c++) {
			run("Grid/Collection stitching", "type=[Filename defined
position] order=[Defined by filename         ] grid_size_x="+stitchX+"
grid_size_y="+stitchY+" tile_overlap="+stitchO+" first_file_index_x=0
first_file_index_y=0 directory="+tmpdir+"
file_names=["+filebase+dataindicator+pmtnames[c]+"{yy} x
{xx}_C"+IJ.pad(c,2)+"_xyz-Table Z"+IJ.pad(z,4)+".ome.tif]
output_textfile_name=TileConfiguration.txt fusion_method=[Linear Blending]
regression_threshold=0.30 max/avg_displacement_threshold=2.50
absolute_displacement_threshold=3.50 subpixel_accuracy
computation_parameters=[Save computation time (but use more RAM)]
image_output=[Fuse and display]");
			run("Properties...", "channels=1 slices=1 frames=1 unit=um
pixel_width="+pxx+" pixel_height="+pxy+" voxel_depth="+pxz);
			saveAs("TIFF", outdir + filebase + "C" + IJ.pad(c,2) + "_Z"
+ IJ.pad(z,4) + ".tif");
		}
		tmplist=getFileList(tmpdir);
```

```
            var ok = 1;
            for (i=0; i<tmplist.length; i++) {
                  ok = File.delete(tmpdir+tmplist[i]);
                  if (ok == 0)
                        IJ.log("Cannot delete temp file"+tmpdir+tmplist[i]);
            }
      }

      setBatchMode(false);
      IJ.log("====== "+filebase+" PMT Data Stitching Completed. ======");
}



macro "-" { } //menu divider



/*
 * This command creates a straigthen image of a freehand line and saves it in
the profiles folder
 * (as set in the preferences above)
 */
macro "Straighten Profile[t]" {
      var profilesdir=call("ij.Prefs.get", "phasor.plotprofilesdir","");
      s = getSliceNumber()-1;
      run("Fit Spline");
      run("Straighten...");
      run("Rotate 90 Degrees Right");
      saveAs("TIFF", profilesdir +  "Plot Profile Z"+IJ.pad(s,4)+".txt");
      run("Select All");
      run("Clear Results");
      profile = getProfile();
      for (i=0; i<profile.length; i++) {
            setResult("Value", i, profile[i]);
      }
      saveAs("Results", profilesdir + "Plot Profile Z"+IJ.pad(s,4)+".txt");
      run("Close");
}



/*
 * Draws a line that fills the left side of a profile image
 */
macro "Draw Half-Line Left[a]" {
      getDimensions(w,h,c,s,f);
      hx=round(h/2);
      wx=round(w/2);
      makeLine(wx,hx,0,hx,h);
}



/*
 * Draws a line that fills the right side of a profile image
 */
macro "Draw Half-Line Right[s]" {
      getDimensions(w,h,c,s,f);
      hx=round(h/2);
      wx=round(w/2);
      makeLine(wx,hx,w,hx,h);
}
```

```
/*
 * Creates consecutive ROIs analysis on a profile image and quantifies
intensity for each region
 * Run after drawing a half line left or right
 */
macro "Analyze NAD+/NADH Profile[d]" {
      inputdir=getDirectory("Profiles directory");

      Dialog.create("Dimensions");
      Dialog.addNumber("Microns", 1, 0, 6, "");
      Dialog.addNumber("Pixels", 1, 0, 6, "");
      Dialog.show();
      microns = Dialog.getNumber();
      pixels = Dialog.getNumber();

      run("Set Measurements...", "mean standard limit display");// mean and
SD, limit to threshold

      profilelist = getFileList(inputdir);
      for (k=0; k< profilelist.length; k++) {
            if(matches(profilelist[k], ".*\\.tif")) {
                  open(inputdir+profilelist[k]);
                  stack=getImageID();
                  title=getTitle();
                  run("Set Scale...", "distance="+microns+" known="+pixels+"
pixel=1 unit=um");
                  run("Clear Results");
                  run("Reslice [/]...", "output=1.000 start=Left
avoid");//1px spacing avoid interpolation
                  reslices=getImageID();
                  selectImage(stack);
                  close();
                  selectImage(reslices);
                  rename("reslice "+title);
                  setThreshold(5,150);
                  for (i=0; i< nSlices; i++) {
                        selectImage(reslices);
                        setSlice(i+1);
                        run("Select All");
                        run("Measure");
                  }
                  saveAs("Measurements", inputdir+"Results "+title+".txt");
                  close();
            }
      }

}



/*
 * Creates consecutive ROIs analysis on a profile image and quantifies
average value for each region
 * Run after drawing a half line left or right
 */
macro "Analyze pH Profile[f]" {
      /* NB Signs are a bit odd to take into account that the origin
       *    is the upper left corner:
       *    y axis = -y
       */
      hMin=0;
      hMax=12;
```

```
      nBins=200;
      nregions=20;
      title=getTitle();
      stack = getImageID();
      getLine(x1, y1, x2, y2, lineWidth);
      if (lineWidth <= 0)
            exit("Please provide a line width (Edit->Selection->Properties...
or Ctrl+Y)");
      linelength = sqrt(pow(abs(x1-x2),2)+pow(abs(y1-y2),2));
      radangle = atan2(y1-y2,x2-x1);
      reglength = round(linelength/nregions);
      roiManager("Reset");
      for ( i = 0 ; i < nregions; i++) {
            xtmp1 = x1+i*reglength*cos(radangle);
            xtmp2 = x1+(i+1)*reglength*cos(radangle);
            ytmp1 = y1-i*reglength*sin(radangle);
            ytmp2 = y1-(i+1)*reglength*sin(radangle);
            makeLine(xtmp1,ytmp1,xtmp2,ytmp2,lineWidth);
            run("Line to Area");
            roiManager("Add");
      }
      n = roiManager("count");
      print("\\Clear");
      for (i=0; i<n; i++) {
            selectImage(stack);
            roiManager("select", i);
            getHistogram(values,counts,nBins,hMin,hMax);
            for (j=1; j<nBins; j++) {
                    IJ.log((i+1)+","+values[j]+","+counts[j]);
              }
      }

}



/*
 * Quantifies intensity profile in profile images
 * Run after drawing a half line left or right
 */
macro "Analyze Hypoxic Profile[g]" {
       run("Plot Profile");
}
```
